# Frontal alpha asymmetry during emotion regulation in adults with lifetime major depression

**DOI:** 10.3758/s13415-024-01165-0

**Published:** 2024-02-01

**Authors:** Carolin Zsigo, Ellen Greimel, Regine Primbs, Jürgen Bartling, Gerd Schulte-Körne, Lisa Feldmann

**Affiliations:** Department of Child and Adolescent Psychiatry, Psychosomatics and Psychotherapy, LMU University Hospital, LMU Munich, Nußbaumstr. 5, 80336 Munich, Germany

**Keywords:** Depression, Emotion, Regulation

## Abstract

Emotion regulation (ER) often is impaired in current or remitted major depression (MD), although the extent of the deficits is not fully understood. Recent studies suggest that frontal alpha asymmetry (FAA) could be a promising electrophysiological measure to investigate ER. The purpose of this study was to investigate ER differences between participants with lifetime major depression (lifetime MD) and healthy controls (HC) for the first time in an experimental task by using FAA. We compared lifetime MD (*n* = 34) and HC (*n* = 25) participants aged 18–24 years in (a) an active ER condition, in which participants were instructed to reappraise negative images and (b) a condition in which they attended to the images while an EEG was recorded. We also report FAA results from an independent sample of adolescents with current MD (*n* = 36) and HC adolescents (*n* = 38). In the main sample, both groups were able to decrease self-reported negative affect in response to negative images through ER, without significant group differences. We found no differences between groups or conditions in FAA, which was replicated within the independent adolescent sample. The lifetime MD group also reported less adaptive ER in daily life and higher difficulty of ER during the task. The lack of differences between in self-reported affect and FAA between lifetime MD and HC groups in the active ER task indicates that lifetime MD participants show no impairments when instructed to apply an adaptive ER strategy. Implications for interventional aspects are discussed.

**Supplementary Information:**

The online version contains supplementary material available at 10.3758/s13415-024-01165-0.

## Introduction

Major depression (MD) is a severe mental illness with debilitating consequences on both cognitive and social functioning (McIntyre et al., 2013). In young adulthood, a period marked by the transition from adolescence into adult life, the prevalence of MD is high, with a 12-month prevalence of 7–9% (Gustavson et al., 2018; Mojtabai et al., 2016). For those who had already suffered from MD earlier in adolescence, there is a strong risk of reoccurrence upon entering adulthood (Alaie et al., 2019; Benjet et al., 2020). Moreover, even after remission, deficits often remain, such as residual depressive symptoms, social dysfunction and dysfunctional attitudes (Saragoussi et al., 2018), or cognitive deficits, such as deficits in selective attention, working memory, and long-term memory (Semkovska et al., 2019).

One domain that is impacted by MD is emotion regulation (ER)—a skill that shows marked development during emerging and young adulthood (Zimmermann & Iwanski, 2014) and which often stays deficient even after remission (Visted et al., 2018). Patients with MD tend to use more ineffective and maladaptive ER strategies in daily life as well as less effective and adaptive ER strategies (Joormann & Stanton, 2016; Zsigo et al., 2023) compared with healthy controls (HC). One adaptive strategy that often is underused or misused in MD is cognitive reappraisal (CR)—a strategy in which negative life events are reinterpreted in a different light by changing the subjective meaning of a situation or an event (Dryman & Heimberg, 2018). As such, individuals with MD, compared with HCs, often use less CR in daily life (for a review, see Visted et al., 2018).

However, when researching CR skills in depression in a structured task, studies often find no difference between patients with MD and HCs in the ability to successfully apply CR.

Such CR paradigms usually present averse or negative stimuli with the instruction to reappraise the aversive content of the stimuli to reduce their emotional intensity. This is contrasted with negative stimuli that are only passively attended to. CR ability is then measured as the contrast of the self-reported negative affect ratings of the reappraised images to the passively attended ones; successful CR displays more positive ratings of reappraised than of attended stimuli.

This study was designed to measure whether participants can successfully reduce negative affect (based on self-report) when using CR. In such paradigms, both adolescents (Feldmann et al., 2022; LeWinn et al., 2018) and adults with current (Dillon & Pizzagalli, 2013; Erk et al., 2010; Kanske et al., 2012; Millgram et al., 2015) and remitted MD (Smoski et al., 2015; Smoski et al., 2013) do not show any differences in their ability to downregulate negative emotions. However, some studies have found that healthy controls show relatively more CR success than MD patients (Greening et al., 2014; Stephanou et al., 2017).

Taken together, questionnaire self-report data show that individuals with MD apply CR less effectively and less often, while experimental paradigms where participants are asked to actively use CR mostly show no differences in self-reported CR success between those with MD and healthy controls.

It should be considered, however, that self-reported data, on which both the habitual and the behavioral results are based, can be biased by a number of factors, such as social desirability or the individual’s ability to perceive their own emotions, which can be impaired in MD (Hemming et al., 2019). Therefore, more objective and sensitive measures of CR are needed to determine the full scope of possible deficits. One method for obtaining more sensitive data of CR deficits can be through electroencephalographic (EEG) measures. In this context, frontal alpha asymmetry (FAA) has been connected to several measures of emotion and ER (Reznik & Allen, 2017).

FAA describes the relative difference in alpha activity between the left and right hemispheres, typically measured via EEG across frontal regions. Alpha activity can be seen as an inverse measure of cortical activity, with a decrease in alpha power reflecting an increase in cortical activity and an increase in alpha power indicating a decrease in cortical activity (Bazanova & Vernon, 2014). In general, relative left frontal cortical activity (rLFA, i.e., more left frontal cortical activity compared with right) has been found to be connected to approach motivation, positive affect, and better ER (for a review, see Reznik & Allen, 2017). Meanwhile, relative right frontal cortical activity (rRFA; i.e., more right frontal cortical activity compared with left) has been associated with withdrawal motivation (Harmon-Jones & Gable, 2018) and both depressive symptoms (Thibodeau et al., 2006) and current and past depression status (for a review, see Allen & Reznik, 2015). It also has been found that rRFA prospectively predicts both higher depressive symptomatology 1 year later (Stewart & Allen, 2018) and a first depressive episode in previously healthy participants (Nusslock et al., 2011). In one study, lifetime MD (i.e., current or remitted) has been linked to rRFA, indicating that this connection is not dependent on MD status (Stewart et al., 2010).

However, all of these studies were conducted during rest, and it should be noted that there have been recent meta-analyses calling this connection between FAA and depression during rest into question (Kołodziej et al., 2021), highlighting the importance of more task-based approaches. Indeed, the so-called capability model of frontal EEG asymmetry proposes that individual differences in FAA might be more pronounced during emotionally evocative tasks, as they reflect the interactions between emotional demands of specific situations and the individual’s own capabilities (Coan et al., 2006). As such, in healthy populations, viewing disaster-related film clips was associated with increased rRFA (Papousek et al., 2014) and higher rRFA under stress was predictive of continued negative emotion in a subsequent task (Pérez-Edgar et al., 2013). In a similar vein, healthy participants with increased rRFA under negative emotion induction tended to show no emotional recovery after the task (Haehl et al., 2020). Taken together, FAA, specifically rRFA, seems to be a consistent marker for negative affective states.

Considering this, FAA could be promising to investigate ER, as a reduction of negative affect through successful ER should then be associated with a reduction in right frontal activity resulting in changes in FAA.

Interesting starting points for the investigation of ER via FAA come from studies in healthy samples. As such, in healthy adults, greater rLFA was connected to less difficulties in habitual ER (Zhang et al., 2020).

Few studies so far have researched FAA in active ER tasks. One study found that individuals who had higher capacity to generate reappraisals showed greater rLFA during a reappraisal task (Papousek et al., 2017). Two studies asked participants to regulate their emotions in response to negative stimuli and found no difference to nonregulation conditions; however, one of them asked participants to employ expressive suppression, i.e., to suppress their emotional response (Lacey et al., 2020), whereas the other did not instruct participants to use a specific ER strategy (Yang et al., 2021). In contrast, in a study that specifically instructed participants to use CR, a decrease in in left-frontal alpha activity following CR (compared with a nonregulation condition) was found (Parvaz et al., 2012). It should be noted that this study did not calculate an asymmetry index and rather investigated each hemisphere separately.

Finally, a study by Choi et al. (2016) found that during reappraisal, participants experienced a shift toward rLFA, which the authors interpret to reflect the decreased emotional response to the negative image when reappraisal is applied. Interestingly, Choi et al. (2016) also repeated their experiment while instructing participants to suppress their emotions, rather than reappraise the images, and found no effect on FAA. The authors concluded that suppression might not be an effective ER strategy and does not lead to changes in affect, whereas CR is better suited to downregulate negative emotions. Taken together, studies suggest that FAA might be a useful measure of CR. However, because there are only few studies researching CR, further research is needed to validate these results.

The studies mentioned above all investigate healthy subjects; to date, there have been no studies examining FAA in the context of ER deficits in MD. Some evidence has studied emotionally evocative tasks in participants with MD or depressive symptomatology: During presentation of emotional film clips, high-risk children (with mothers with a history of MD) demonstrated more rRFA than low-risk children (Lopez-Duran et al., 2012). It also was found that women with premenstrual dysphoric disorder, both when depressive mood was inducted and during a relaxation period afterwards, showed significantly more rRFA than women without the disorder (Lin et al., 2013). During an emotional imagery task involving images, dysphoric individuals also showed increased rRFA compared with nondysphoric individuals (Mennella et al., 2015). After experiencing rejection, participants with MD also displayed more rRFA than healthy participants (Beeney et al., 2014). Finally, in a large sample of participants who were instructed to make approach (angry or happy) and withdrawal (afraid or sad) facial expressions, those with lifetime MD displayed increased rRFA compared with HCs (Stewart et al., 2011). There seems to be evidence that during emotional tasks, MD also is associated with increased rRFA.

Therefore, based on the association between active CR and increased rLFA both habitually (Zhang et al., 2020) and during active CR in an experimental paradigm (Choi et al., 2016; Parvaz et al., 2012), we would expect that during reappraisal compared with passively attending negative images, healthy controls would have an increase in rLFA. Taking a step further, based on 1) the fact that an increase in rRFA is associated with lifetime MD, specifically during emotionally evocative tasks (Allen & Reznik, 2015; Stewart et al., 2010; Stewart et al., 2011), and 2) MD is associated with deficits in habitual ER (Visted et al., 2018), we would expect that the reappraise-specific increase in rLFA would be smaller in participants with lifetime MD.

To investigate this, we employed a CR task with two conditions, in which participants were instructed to either (1) reappraise negative pictures by imagining a different, more positive, interpretation of the shown image, thereby changing the subjective meaning (Denny & Ochsner, 2014) or (2) attend to negative pictures without changing their emotional response. After each picture, we asked participants to rate their emotional response to the shown picture on a valence scale.

On a self-report level, we expected that both groups would rate images more positively after reappraisal of the images, without significant group differences (Millgram et al., 2015; Smoski et al., 2013). Because this is the first study to research FAA in an active ER paradigm in the context of MD, we also report FAA results from a separate study in currently depressed adolescents compared with a healthy control group of adolescents.

## Methods

### Participants

In the present study, participants with lifetime MD (*n* = 34), that is, participants who had either a current or past diagnosis of MD, and HCs (*n* = 25) between the ages of 18–24 years were included. Details on sample size calculation can be found in Supplement A.

All participants in the lifetime MD group and 18 HCs were recruited from a pool of participants who had previously participated in a study on child and adolescent major depression at the Department of Child and Adolescent Psychiatry, Psychosomatics and Psychotherapy at the LMU Hospital in Munich 5 years ago. An additional seven HCs were recruited via flyers and the department’s website. Participants were recruited between February 2021 and April 2022. All procedures were approved by the ethics committee of the LMU Hospital, and all participants were informed about the procedures and goals of the study and provided written, informed consent. As compensation for their participation, participants received 50€ vouchers.

To be included in the study, participants had to have an intelligence quotient (IQ) ≥ 85, as measured by the CFT-20-R (Culture Fair Intelligence Test; Weiß, 2019) or other established IQ measures, such as the WIE (Wechsler Adult Intelligence Scale, German: "Wechsler Intelligenztest für Erwachsene"; von Aster et al., 2006). Participants were only included in the HC group if they did not meet any ICD-10 (World Health Organization, 1992) criteria for current or lifetime diagnoses of any psychiatric disorder according to the DIPS (Diagnostic Interview of Psychiatric Disorders, German: “Diagnostisches Interview psychischer Störungen”), which is a well-established, German, semistructured, clinical interview (Margraf et al., 2017; Schneider & Margraf, 2005). All HCs had a BDI-II (Beck’s Depression Inventory II) score ≤ 4, which corresponds to no depression according to the BDI-II manual (Hautzinger et al., 2006).

To be included in the lifetime MD group, participants had to fulfill criteria for lifetime major depressive disorder according to the classification specified in the ICD-10 (World Health Organization, 1992), as measured by the DIPS (Margraf et al., 2017; Schneider & Margraf, 2005). Participants with comorbid lifetime bipolar disorder, schizophrenia, and pervasive developmental disorder were excluded. Other comorbidities in the lifetime MD group were accepted. In the lifetime MD group, 17 participants showed a current comorbidity, most of which were anxiety disorders. At the time of the EEG recording, nine participants received psychopharmacological medication (7 of those received an SSRI, 1 an SNRI, and 1 a MAOI). Exclusion of these participants did not change the patterns of our results, so findings below include participants with active medication. Groups were comparable regarding sex and age. Demographics and test statistics are shown in Table 1. Demographics and differences between remitted and current MD participants of the lifetime MD group of the main sample can be found in Supplement B: Table 1.

**Table 1:** Sample characteristics

	Lifetime MD (n = 34)	HC (n = 25)	Test	
			*t*	*p*
Age in years (*M, SD*)	21.06 (1.61)	20.52 (1.73)	1.228	0.224
Age range	18-24	18-23		
Sex (% female)	76%	85%	0.820^a^	0.365
BDI-II score (*M, SD*)	14.21 (11.50)	2.64 (2.30)	4.946	<.001
ERQ reappraisal (*M, SD*)	23.29 (7.35)	29.72 (5.08)	−3.757	<.001

*MD* = major depression; *HC* = healthy control; *M* = mean; *SD* = standard deviation; *BDI *= Beck’s Depression Inventory; *ERQ* = Emotion Regulation Questionnaire

^a^Pearson’s chi-square statistic

### Materials

Self-reported depressive symptomatology was assessed with the German version of the BDI-II (Beck’s Depression Inventory, Second Edition; Hautzinger et al., 2006). In our sample, internal consistency was excellent (Cronbach’s α = .95). Self-reported CR skills were assessed with the German version of the ERQ (Emotion Regulation Questionnaire; Abler & Kessler, 2009). The ERQ contains six items measuring reappraisal, with each item rated on a 7-point Likert scale ranging from 1 (strongly disagree) to 7 (strongly agree), e.g., “When I want to feel more positive emotion, I change the way I’m thinking about the situation.” Internal consistency for ERQ reappraisal in our sample was good (Cronbach’s α = .80). As can be expected, the lifetime MD group showed significantly higher BDI-II scores than the HC group, as well as significantly lower ERQ reappraisal scores (Table 1).

### Experimental procedure

The experimental task used was adapted from established paradigms (Denny & Ochsner, 2014; Paul et al., 2013; Schönfelder et al., 2014) and has been successfully employed to measure CR in MD before (Feldmann et al., 2022; Greimel et al., 2020; Piechaczek et al., 2022). Participants were invited to two sessions: one diagnostic session (approx. 1-2 hours), in which the DIPS was applied, and one experimental session, in which the CR task (approx. 1.5–2 hours) was conducted as described below. All sessions were performed by one experimenter with a master's degree in clinical psychology.

#### Training and practice trials

Before the start of the experiment, participants were instructed by the experimenter. They were told that they would be shown neutral, positive, and negative images, which would be preceded by a specific instruction (“attend” or “reappraise”). They were told that during the attend condition, their task would be to attentively view the picture and respond naturally to it without trying to influence their initial emotional reaction, independent of whether the image was neutral, positive, or negative. During the reappraise condition, which only contained negative images, they were instructed to attentively view the image and then decrease their initial negative emotional reaction through the use of CR. Specifically, they were trained to use the CR strategy reinterpretation, in which they were told to change the subjective meaning of the event by imagining a more positive outcome or choosing a more positive interpretation of the situation (Denny & Ochsner, 2014). For example, when presented a photo of an accident, they could imagine that no one was severely hurt or that help is already on the way (Ochsner et al., 2012).

After the instruction, participants were given a walk-through on three different reappraisal practice images, during which they applied CR under the guidance of the experimenter, as well as two attend practice images. They also were trained to rate their emotional response to each image on a 9-point Self-Assessment Manikin (SAM) rating scale (Bradley & Lang, 1994; with the portrait version from Lang, 1980; Suk, 2006) from 1 (strongly negative) to 9 (strongly positive). Finally, participants were presented 12 practice trials (6 attend, 6 reappraise) on the experimental computer. Participants could turn to the experimenter at any point during the practice trials if they had questions.

#### Experimental trials

Participants were shown 144 experimental trials. These were separated into three blocks, each of which contained all four experimental conditions, which were presented in a randomized order: 1) negative-reappraise; 2) negative-attend; 3) neutral-attend; 4) positive-attend. Each condition contained 12 consecutive trials, with the images therein also presented in a randomized order to avoid a potential task switching effect. Each image was assigned to exactly one condition, so that, in the negative conditions, 36 images were attended to and 36 different images were reappraised. While the focus of this study lies on the negative-reappraise and negative-attend conditions, the neutral and positive conditions were added to avoid negative mood induction over the course of the experiment. Each block lasted approximately 15 min, followed by a short break (~5 min each).

**Fig. 1:**
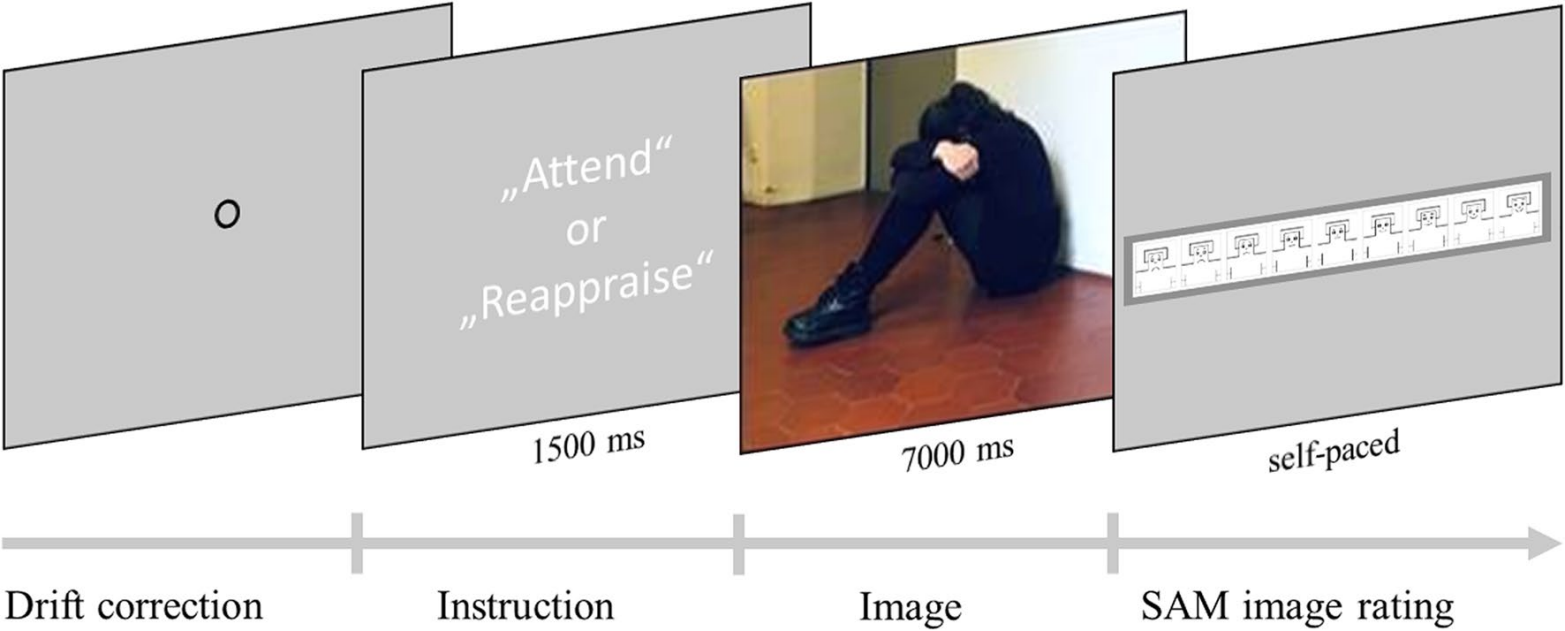
Trial structure. Image is an example and not part of the IAPS database

The time course of a trial is illustrated in Fig. 1. At the start of each condition, participants were given an “attend” or “reappraise” cue. During the experiment, participants were seated in front of an Eyelink 1000 Plus Desktop Mount Eye-Tracker, which started each trial with a drift correction. The eye-tracking data will not be presented in this manuscript. After drift correction, an instructional cue reminded the participant of the strategy they should apply (“attend” or “reappraise”) for 1.5 s. Afterwards, an image was presented for 7 s. Finally, participants were shown the 9-point SAM valence rating scale (Bradley & Lang, 1994; with the portrait version from Lang, 1980; Suk, 2006).

After the experiment, participants were given a self-developed questionnaire to ask the participants how hard it was for them to follow the two negative experimental conditions (negative-attend and negative-reappraise) as well as to separate the two strategies: scale from 1 (“very easy”) to 5 (“very difficult”). They also entered freeform answers describing what they did during each condition in their own words to make sure they understood the instructions as intended. All participants described how to apply the strategies correctly. There was no difference between the lifetime MD (*M* = 1.91, *SD* = 0.93) and the HC (*M* = 1.79, *SD* = 0.78) groups when rating how difficult it was to apply the negative-attend instruction (*t*(56) = 0.52, *p* = .608). However, the lifetime MD group (*M* = 3.21, *SD* = 0.81) found it significantly harder than the HC group (*M* = 2.46, *SD* = 0.83) to apply the negative-reappraise instruction (*t*(56) = 3.42, *p* = .001). The groups (lifetime MD: *M* = 1.85, *SD* = 0.96; HC: M = 1.75, SD = 0.79) did not differ in how difficult they found it to separate the two conditions from one another (*t*(56) = 0.43, *p* = .668).

#### Stimuli

The 144 images that were used in the present study were taken from the IAPS (International Affective Picture System; Lang et al., 2008) and the BAPS (Besançon Affective Picture Set, Adolescent and Adult versions; Szymanska et al., 2019; Szymanska et al., 2015). Thirty-six images were selected for the neutral and positive conditions each, as well as 72 negative images in total for the two negative conditions. Images were selected to be more likely to induce top-down generated emotions (i.e., emotions generated through the cognitive evaluation of the depicted scenario) than bottom-up generated emotions (i.e., emotions elicited through the inherent properties of the stimulus), because it has been shown that CR can be applied more effectively on such top-down generated responses (McRae et al., 2012).

Images were selected to be comparable between negative-reappraise and negative-attend conditions in luminance (*t*(70) = 1.30, *p* = .20) and whether an image depicted a social or non-social scene (χ^2^(1, 72) = 0, *p* = 1). In a pilot study, a separate sample of participants (*n* = 16) rated arousal (*t*(70) = 0.31, *p* = .756) and valence (*t*(70) = 0.11, *p* = .917) of the negative images, both of which were kept comparable upon the assignment of the images to the two study conditions as well. A detailed list of which IAPS and BAPS pictures were used can be found in Supplement B: Table 2.

### EEG recording, preprocessing, and analysis

**Fig. 2:**
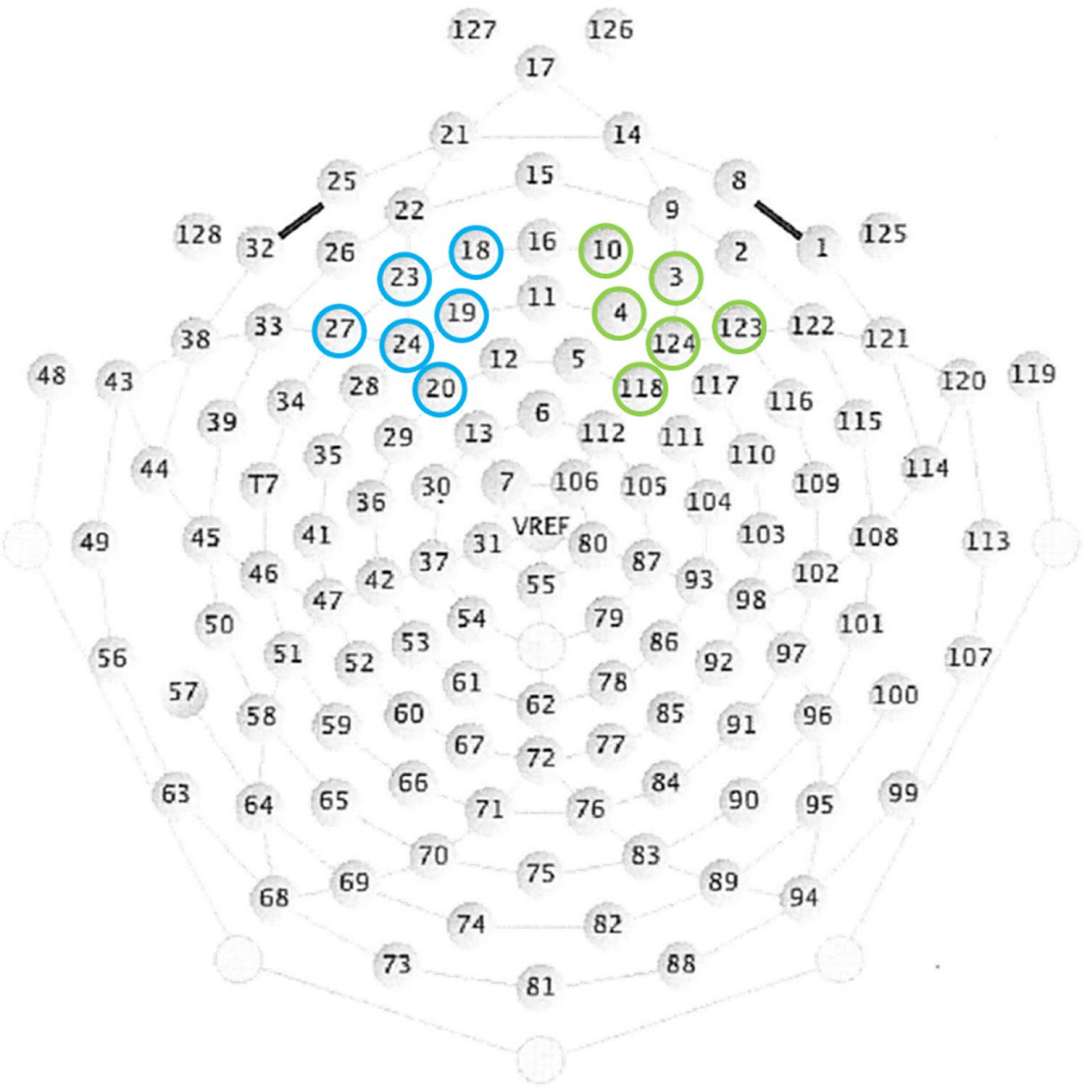
Sensor layout of the Electrical Geodesics Inc. 128-channel system with the right ROI marked in green and the left ROI marked in blue

EEG data were recorded with an Electrical Geodesics Inc. 128-channel system, using a sampling rate of 500 Hz. Sensor layout is shown in Fig. 2. During recording, Cz was used as the reference electrode and impedance in all electrodes was held under 50 kΩ. Data were preprocessed and analyzed with BrainVision Analyser, version 2.2 (Brain Products GmbH, Gilching, Germany). In the case of a faulty electrode, the channel was interpolated using the signal from surrounding electrodes. On average, of 128 electrodes, 2.93 electrodes (2.29% of all electrodes) were interpolated per participant.

Continuous raw data were filtered with an eighth order IIR Butterworth filter with a low cutoff of 0.16 Hz, a high cutoff of 40 Hz, a notch filter of 50 Hz, and a 47 dB/oct roll-off. Artefacts were removed through visual inspection with a nonautomatic Independent Component Analysis (ICA), which was performed by a trained person who was not blind to group, although analysis was performed on pseudonymized data for which group assignment was not directly apparent during data analysis. On average, 34.88 of 128 components were removed per participant in the lifetime MD group and 35.64 of 128 on average per participant in the HC group. Within these components, electro-oculographic (EOG) artefacts, cardiac artefacts, electrodermal, and other nonocular muscular activity were represented and thus removed. It should be noted that, because of the nature of the task, participants had to perform constant eye movement to view the images, and that, because of to the concurrent eye-tracking, participants also had increased muscle tension from laying their head on a chin-rest during the experiment, which can explain why a relatively high number of components had to be removed. Importantly however, the number of components removed did not differ between groups (*p* = .803).

Following ICA, all further analyses were only performed on channels within the two regions of interest relevant to the study, on the left (electrodes 18, 19, 20, 23, 24, 27) and right hemisphere (electrodes 3, 4, 10, 118, 123, 124). These ROIs have been previously used to assess FAA on a 128-channel system (Feldmann et al., 2018; Gabard-Durnam et al., 2015). Placement of ROIs also is shown in Fig. 2.

Artefacts remaining after ICA were removed in individual channels with following thresholds: gradient max 40 µV/ms; max-min 200 µV/ms for 200 ms windows; max amplitude 150 µV; min amplitude −150 µV; low activity 0.5 µV for 100 ms windows (see also Feldmann et al., 2018). Only participants with at least 80 s of recording remaining after artefact removal per experimental condition were included in the analysis. A minimum of 80 s of artifact-free recorded data was shown to be sufficient to achieve good reliability of FAA data (Towers & Allen, 2009). No participant had to be excluded due to this criterion. In Supplement B: Table 3, an overview of how many segments were included on average per group per condition, as well as what percentage of data was removed because of artifacts remaining after ICA, can be found.

After artefact removal, the reference was then replaced with the reference-free current source densities (CSD). The use of the reference-free CSD is recommended in the assessment of FAA in participants with depressive disorders, as it has been shown to be able to accurately differentiate between participants with lifetime MD and never-depressed participants (Smith et al., 2017; Stewart et al., 2010). The calculation of the CSD was based on the spherical spline model (Perrin et al., 1989, 1990) with the following parameters: order of splines = 4; maximal degree of legendre polynomials = 20 (Feldmann et al., 2018; Kamarajan et al., 2015).

Data were then segmented into the four experimental conditions: negative-reappraise, negative-attend, positive-attend, and neutral-attend. Data were further separated into 2.048 s segments with 50% segment overlap, separately for each condition. A Fast Fourier Transformation was applied to obtain spectral power at a resolution of 0.5 Hz with a Hanning window (for a similar approach, see Smith et al., 2017). Groups did not differ in the number of segments included in any of the conditions (*ps* > .675).

Alpha spectrum was defined as 8–13 Hz, actual frequency resolution of the final spectrum was 0.48 Hz. After applying natural logarithmic transformation, values were averaged separately across the left and right ROIs.

### Independent adolescent sample

To validate our main findings concerning FAA, we have conducted the same analyses as detailed below on a separate sample of adolescents. FAA data of these adolescents were taken from a larger project on emotion regulation and MD in adolescence, in which another electrophysiological measure of ER (the late positive potential) was analyzed (Feldmann et al., 2023).

In total, the independent sample consisted of adolescents with current MD (*n* = 36) and adolescents as healthy controls (HC, *n* = 38) between the ages of 12–18 years; 79.7% of the sample were female. The mean age was 15.50 years (*SD* = 1.57). Inclusion and exclusion criteria were the same as for the main sample; the only difference was that psychiatric diagnoses were evaluated with the child and adolescent version of the DIPS (Kinder-DIPS; Margraf et al., 2017) and that in some cases, an intelligence test for children and adolescents was used to measure IQ (e.g., the Wechsler Intelligence Scale for Children, WISC-IV or WISC-V) instead of applying the CFT-20. The ER experiment, stimuli, EEG recording, preprocessing, and analysis were equal to those described for the main sample. For the independent adolescent sample, the average number of interpolated electrodes per participant was 2.29 of 128 electrodes (1.79%). The average number of channels removed during ICA was 16.61 in the HC and 18.50 in the MD group of 128 components, which does not differ significantly between groups (*p* = .195). The number of segments and percentage of data removed because of artefacts in the independent sample can be found in Supplement B: Table 4. In the independent adolescent sample, the main statistical analyses pertaining to the FAA were repeated as described in the statistical analysis section below.

### Statistical analysis

Statistical data analyses was performed in IBM SPSS Statistics version 29.0.0.0. As the focus of our EEG analyses was on the contrast between the negative-reappraise and the negative-attend conditions, those two were directly compared.

Alpha asymmetry was measured by calculating a laterality index (ln[right ROI]-ln[left ROI]) for each participant (see also Feldmann et al., 2018; Stewart et al., 2010), on which positive scores represent greater left-frontal brain activity than right and negative scores represent greater right frontal brain activity than left. To examine whether the lifetime MD group would show a difference in FAA to the HC group during active CR in comparison to passive viewing of negative images, a 2 (group) x 2 (condition) repeated measures ANOVA was performed. In case of significant interactions, follow-up *t*-tests were conducted. In explorative analyses in Supplement C, we also calculated the same analysis with all four experimental conditions and the same analysis on both study samples (young adults and adolescents) combined. Results for this also can be found in Supplement C.

For the behavioral measure of ER success, i.e., the SAM rating of the negative images, we calculated a 2 (group) x 2 (condition) ANOVA. In case of a significant main effect of condition or a significant interaction, results were followed up with *t*-tests. Again, explorative analyses including all four experimental conditions can be found in Supplement C. It should be noted that, because of technical difficulties, the behavioral data of three participants could not be analyzed, so the behavioral analyses of the SAM ratings are based on 56 participants (HC: *n* = 25, lifetime MD: *n* = 31).

To conduct correlational analyses with FAA scores, we calculated an FAA ER success index (FAA laterality index negative-reappraise minus FAA laterality index negative-attend). This FAA ER success index difference was correlated with an analogous SAM difference score (SAM rating negative-reappraise minus SAM rating negative-attend) and with the ERQ reappraisal score, separately for both groups (for a similar approach, see Choi et al., 2016). Finally, within the lifetime MD group, we correlated the BDI-II score with the FAA ER success index score. Alpha level for all comparisons is set at .05.

## Results

### Laterality index

**Fig. 3.**
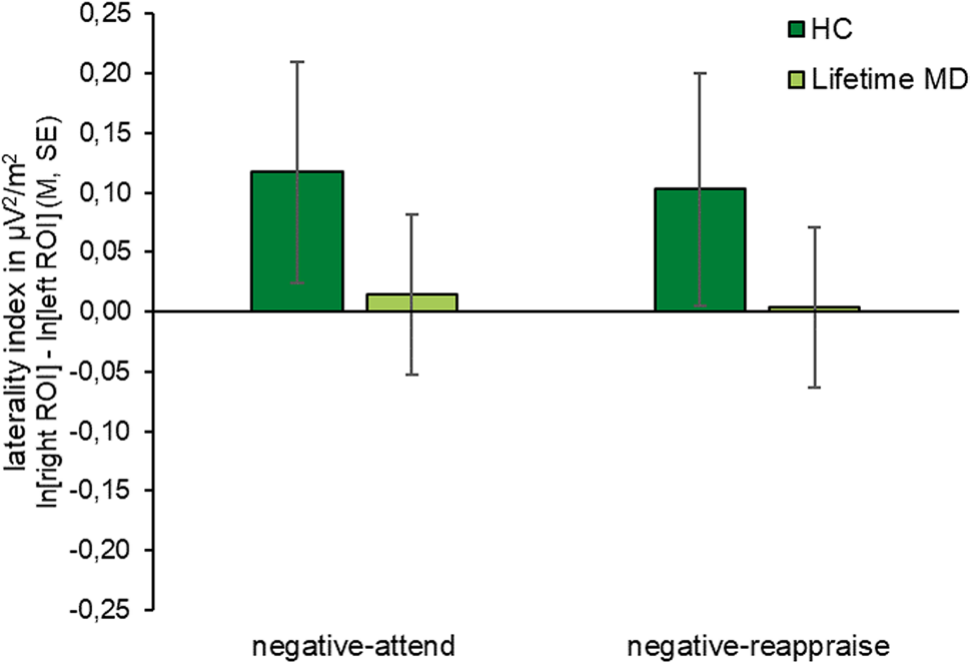
Means and standard errors for the laterality index (ln[right ROI] – ln[left ROI] alpha activity) in µV^2^/m^2^ for both groups in the negative-attend and negative-reappraise conditions

In the 2 (group) x 2 (condition) ANOVA examining the laterality index, there was no main effect of group (*F*(1, 57) = 0.814, *p* = .371, η^2^_*p*_ = 0.014) or condition (*F*(1, 57) = 0.760, *p* = .387, η^2^_*p*_ = 0.013), and no interaction between group*condition (*F*(1, 57) = 0.018, *p* = .894, η^2^_*p*_ < 0.001). Means and standard errors of the laterality index can be found in Fig. 3 as well as in Supplement B: Table 5.

### Self-reported affect in experiment

The 2 (group) x 2 (condition) ANOVA on the SAM image ratings revealed no main effect of group (*F*(1, 54) = 0.760, *p* = .387, η^2^_*p*_ = 0.014) but showed a main effect of condition (*F*(1, 54) = 188.397, *p* < .001, η^2^_*p*_ = 0.777) in that the negative-reappraise images (*M*_lifetime MD_ = 5.05, *SD*_lifetime MD_ = 0.69; *M*_HC_ = 4.89, *SD*_HC_ = 0.66) were rated more positively than the negative-attend images (*M*_lifetime MD_ = 3.29, *SD *_lifetime MD_ = 0.67; *M*_HC_ = 3.20, *SD*_HC_ = 0.71). There was no interaction between group and condition (*F*(1, 54) = 0.055, *p* = .815, η^2^_*p*_ = 0.001).

### Correlations

There were no significant correlations between the FAA ER success index (negative-reappraise – negative-attend) and the SAM image rating difference score (negative-reappraise – negative-attend) within the HC (*r*(24) = 0.175, *p* = .402) or the lifetime MD group (*r*(30) = 0.251, *p* = .173). When correlating the FAA ER success index with the ERQ reappraisal score, similarly, no significant correlations were revealed in the HC group (*r*(24) = 0.323, *p* = .116) or in the lifetime MD group (*r*(33) = -0.131, *p* = .459).

Finally, we correlated the FAA ER success index and the BDI-II within the lifetime MD group. Because the BDI-II was nonnormally distributed, we calculated Spearman’s rho, as recommended when the data is not normally distributed (Bishara & Hittner, 2012). We found no significant correlation between the two measures (Spearman’s *r*(33) = −0.186, *p* = .293).

### FAA in the independent adolescent sample

In the 2 (group) x 2 (condition) ANOVA examining the laterality index, there was no main effect of group (*F*(1, 72) = 1.289, *p* = .260, η^2^_*p*_ = 0.018) or condition (*F*(1, 72) = 1.390, *p* = .242, η^2^_*p*_ = 0.019), and no interaction between group*condition (*F*(1, 72) = 1.277, *p* = .262, η^2^_*p*_ = 0.017).

## Discussion

This is the first study to examine FAA differences during an active ER task in MD. The lifetime MD and HC groups did not significantly differ from one another in FAA. This finding was validated in an independent sample of currently depressed adolescents in comparison to a healthy control group. On a self-report level, participants were able to downregulate their negative affect through CR, irrespective of group.

Our main hypothesis was that the lifetime MD group would show a smaller increase in rLFA compared with the HC group when reappraising compared with the attend condition. However, we did not find any significant differences between the two conditions across the two study groups, a finding that was confirmed by an independent sample of the same task in adolescents with MD.

One explanation about why there are no neurophysiological differences between reappraising and attending has been previously brought up by Yang et al. (2021). The authors argue that a reason for their lack of group differences could be that their stimuli were of moderate emotional intensity to be suitable for an adolescent population and that they might not have been evoking enough to result in differences between the regulation and nonregulation conditions (Yang et al., 2021). In our study, we also excluded highly intense stimuli for ethical reasons, such as images depicting extreme violence, because the task also was applied in adolescents in our independent sample. We chose stimuli that induced top-down–generated emotions rather than bottom-up–generated emotions; as such, top-down emotion-inducing images are thought to be easier to reappraise (McRae et al., 2012). However, the selection of these stimuli might have led to the general emotional intensity of the stimuli being lower, because it has been previously found that top-down negative stimuli can elicit less negative affect than bottom-up negative stimuli (Ochsner et al., 2009). A study by Goodman et al. (2013) also found that participants with higher state FAA exhibited greater emotion regulation but only under sufficient stress (in their case, threat of shock). Therefore, the lack of differences between the study conditions could be explained by the low emotional intensity of our stimuli, which could have failed to elicit enough negative affect and subjective stress.

A second possibility could be that differences were less apparent, because our participants were given different images to reappraise and to attend, whereas Choi et al. (2016) used the same negative images for both their observe and reappraise conditions. This could lead to larger differences in the perception of these images, as the contrast between reappraising and attending could be more apparent to the participant if they applied both conditions to the same image. Finally, there is some heterogeneity between the studies when it comes to the EEG reference. Choi and colleagues (Choi et al., 2016) used the mastoids, Parvaz and colleagues (Parvaz et al., 2012) averaged electrical activity, while we used CSD. CSD has been recommended for FAA in recent work (Smith et al., 2017), but it cannot be ruled out that different reference schemes could lead to diverging results. Nevertheless, the results of our study reveal a need for replication to determine whether differences between active CR and passive viewing can be reliably detected by FAA, specifically if there is a difference between stimuli of higher and lower intensity and, connectedly, under high and low conditions of stress.

This line of discussion makes it apparent that there is a disconnect between participants’ electrophysiological responses, in which conditions did not differ, and subjective responses, in which participants rated reappraised images consistently more positive than attended images. First, it is possible that the self-reported image ratings are influenced by social desirability effects (Zilverstand et al., 2017). Participants received extensive instruction how to apply CR before the task and therefore were aware that reappraisal was supposed to change their affective response to the stimuli, which could have biased their answers in the subsequent valence ratings.

Another possibility is that the neurophysiological and the subjective ratings measure different processes of CR. As Bautista et al. (2022), who found a similar disconnect between their neurophysiological CR measure, the late positive potential (LPP), and participants’ valence ratings, write: “Subjective ratings are more downstream from electrocortical response: ratings are made after picture offset, and reflect a number of intervening, higher order cognitive processes that may not be evident in psychophysiological responses that are more proximal to stimulus presentation” (p. 169).

Finally, it needs to be considered that affect ratings as they are usually employed in ER experiments, on a scale from negative to positive valence, might be too simple to capture the complexity of emotion and emotion regulation. As Walle and Dukes (2023) have recently criticized, categorizing different qualities of emotions, such as anger, sadness, or fear into one “negative” category, might trivialize emotional experiences. Future studies could benefit from a more in-depth measurement of affective responses, taking care to include emotion quality and intensity rather than only valence.

It should be noted that the pattern of results, including the lack of neurophysiological differences despite the presence of difference in valence ratings, is consistent across the two study groups. Interestingly, this is the case even though the lifetime MD group compared with the HC group reported less use of reappraisal in daily life, as measured by the ERQ, which is in line with several established findings (Aldao et al., 2010). Possible theories for this divergence could be that in daily life, individuals with MD lack knowledge about available adaptive ER strategies, such as CR (for a review, see Yoon & Rottenberg, 2020). It also has been found that when not instructed to use a specific ER strategy during negative mood induction, individuals with a lifetime MD tend toward suppressing their negative emotions more often, but when asked to reappraise they showed the same proficiency as never-depressed controls (Ehring et al., 2010). Thus, it seems that in the context of MD, an underutilization of strategies, such as CR, is more at fault for the deficits in self-reported ER than an ineffective utilization (Dryman & Heimberg, 2018). Contributing to this divergence could be the belief of MD individuals that their own emotions are unable to be controlled and thus the attempt to do so would be futile (Yoon & Rottenberg, 2020). This could add to their subjective feeling of reappraisal being difficult to implement, as shown by the reported difficulty of the reappraisal condition being higher in the lifetime MD group than the HC group.

In addition to the main sample, we included an independent sample of adolescents with current MD compared with healthy adolescents in the study. In this sample, we were able to replicate the absent differences between groups and conditions in FAA results of the main sample. The independent adolescent sample was part of a larger project on ER and additional results have been previously published elsewhere (Feldmann et al., 2023). Similar to the findings in the main sample of the current study, adolescents with MD reported less adaptive ER abilities compared with the healthy control group in daily life. However, as described in Feldmann et al. (2023), adolescents with current MD report less self-reported ER success during the task than the healthy adolescents. This stands in contrast to our main sample, where we find no difference in self-reported ER success between groups. There are two main differences between the two samples: the independent sample is a youth sample with current depression, so both developmental stage and current mood state are different from the main sample. There is some research suggesting that aspects of adolescent depression may be specific to that developmental period, such as an increased sensitivity to sadness or a reduced perception of happy affect (Nyquist & Luebbe, 2020), both of which could influence self-reported affect ratings. At the same time, current versus remitted depression could be a contributing factor, because an exacerbation of symptoms might influence participants’ perceptions of their own emotions (Visted et al., 2018). Future studies should include participants from different age groups and mood states to disentangle the effects of mood state and age differences in detail.

Looking at the bigger picture of our results, the question needs to be considered whether FAA is a suitable measure for CR, as well as for differences in CR between HCs and lifetime MD participants. While the connection between negative affect and FAA is well established (Haehl et al., 2020; Papousek et al., 2014; Pérez-Edgar et al., 2013), studies looking at FAA during active ER tasks have been mixed. In their discussion, Lacey et al. (2020) argue that FAA might relate more to individual differences (i.e., in behavior or personality) rather than differences in study conditions (Lacey et al., 2020). Indeed, there are studies that have found differences in FAA during active ER between, e.g., individuals with high and low mindfulness (Deng et al., 2021) or with high or low levels of schizotypy (Pan et al., 2020). In addition, there have been findings that FAA can be influenced by cognitive factors, such as working memory load and may be less accurate in tasks requiring higher amounts of concentration (Briesemeister et al., 2013; Grissmann et al., 2017). Thus, future studies should look not only at broader condition or group differences but also at individual differences and possible confounding factors, such as working memory load, to further inform this field of research.

### Strengths and limitations

An important strength of our study is the use of an established experimental task, which has been successfully employed to measure ER in the past (Feldmann et al., 2022; Greimel et al., 2020; Piechaczek et al., 2022). We also recruited a homogeneous sample of young adults, an age group that is of high relevance in research into psychopathology, such as MD (Schulenberg et al., 2004), and confirmed main findings on FAA in an independent sample of adolescents. We also employed standardized diagnostic measures to determine inclusion criteria and depression status in both samples. Finally, we were able to measure habitual ER, self-reported affect, and neurophysiological responses to look at subjective and objective measures of ER at the same time. We also collected continuous eye-tracking data, which, while we do not report results in this manuscript, also are an important part of a multimodal approach; especially pupillary responses might prove an important measure to be considered in future studies (Yang et al., 2023).

There are a number of limitations to consider. In both the main and the independent sample, we included participants with current comorbidities in the MD groups. Studies have found that FAA patterns can be divergent if other disorders are present (Feldmann et al., 2018; Ischebeck et al., 2014; López-Castro et al., 2021); however, as comorbidities reflect the reality of any clinical sample, it is important to include them in research on MD. It should be noted that, because of artefacts, a larger amount of ICA components had to be removed in our main study sample. To our knowledge, there are no official guidelines about how many components should be excluded during the ICA. Although we replicated our FAA results in an independent sample of youths where we excluded less ICA components, it should be noted that removal of a large amount of ICA components can impact on study results. Finally, while we did ask participants to describe how they applied the task conditions after they were finished with the experiment, we did not ask participants to describe their reappraisals during the task. Because studies have found FAA differences between participants who were better at generating creative reappraisals of negative stimuli compared with those less able to do so (Papousek et al., 2017), it would be interesting to include such a measure in the future.

## Conclusions and Future Directions

The findings of this study show that young adults with lifetime MD compared with HCs report to use less CR in daily life and higher difficulty when instructed to apply CR but do not differ in ER success on a self-report or neurophysiological level during an active ER task compared with never-depressed HCs. The neurophysiological findings were confirmed in an independent sample of currently depressed adolescents. Taken together, it seems that there is a gap between the ER abilities of lifetime MD participants and their own perception of it. Taking this into account, treatment of MD could benefit from focusing on the selection of helpful ER strategies or awareness about own ER abilities rather than trying to improve ER skills on their own. Future studies should examine whether perception of one’s ER abilities has an impact on the performance in ER tasks and whether individual differences can be more of an influence on FAA than group or condition differences. In this vein, it would be useful to research how conditions of stress impact CR, specifically whether effects differ depending on stimulus intensity.

Finally, future studies should consider measuring emotion quality alongside simpler valence ratings to depict a wider range of emotional experiences in subjective ratings and to apply longer follow-up measurements to determine whether CR has more long-term effects on neurophysiological measures.

### Supplementary Information

Below is the link to the electronic supplementary material.

Supplementary file1 (DOCX 49 KB)

## Data Availability

Nonaggregated data in our study contain sensitive patient information, such as information on comorbidities. Because patients could possibly be identified by making our raw data publicly available, ethical principles of protecting patient confidentiality would be breached. Therefore, raw data cannot be made publicly available. Additional materials and aggregated data, however, can be made available upon request.
