# Supplementary material for: Frontal alpha asymmetry during emotion regulation in adults with lifetime major depression
Source: Cogn Affect Behav Neurosci. 2024 Feb 1;24(3):552–66. doi: 10.3758/s13415-024-01165-0 (PMC11078823; doi:10.3758/s13415-024-01165-0)
Supplement: Supplementary file 1 — Supplementary file1 (DOCX 49 KB) [file 13415_2024_1165_MOESM1_ESM.docx]

# Supplement

# A: Sample Size Calculation

To our knowledge, this study is the first to research FAA differences between lifetime MD and HC adults in an active ER task, where a reappraisal and an attend condition are compared. Looking at frontal alpha asymmetry studies in MD or MD-adjacent samples (i.e. dysphoria or at-risk populations) in general, there are a number of studies comparing different emotion conditions (e.g. presentation of emotional images or emotion induction). An overview of the studies is presented in the table below. They generally find no significant emotion x group (depression diagnosis) interactions, however, it needs to be considered that emotional vs. non-emotional stimuli are very different from a regulation vs. a non-regulation condition, as the latter calls upon the participant to actively influence their thoughts and affect. We were therefore unable to draw upon prior studies to calculate the necessary sample size to detect our condition (attend, reappraise) x group (lifetime MD, HC) interaction. In order to nonetheless gain an estimate for our sample size, we looked at the post-hoc comparisons that would follow from a significant interaction, specifically the difference between the two groups in the negative-attend condition, as this condition is the closest to the emotional conditions previous studies have employed. Generally, these studies find moderate to large effect sizes (d = 0.5 to d = 1.1) for the difference between MD or MD-adjacent samples and HCs. We would therefore also expect a moderate to large effect for the group difference in the negative attend condition (d ≈ 0.7), which would correspond to a total sample size of 52 participants as calculated by G*Power 3.1.2 (Faul et al., 2009; Faul et al., 2007), assuming α = 0.05 and 1-β = 0.80. Moreover, as other studies that research regulation vs. non-regulation conditions in other contexts (e.g. on the late positive potential or in fMRI studies; Belden et al., 2015; Kudinova et al., 2016; Yuan et al., 2023) generally find greater differences between groups in the regulation conditions, we argue that this number of participants is also enough to detect group difference in the negative-reappraise condition. The studies referenced for these effects are also listed in the table below.

| Study | Sample / Paradigm | Suitability / Results |
| --- | --- | --- |
| **Frontal Alpha Asymmetry Studies in MD / MD-adjacent Samples** | | |
| Mennella, R., Benvenuti, S. M., Buodo, G., & Palomba, D. (2015). Emotional modulation of alpha asymmetry in dysphoria: results from an emotional imagery task. International Journal of Psychophysiology, 97(2), 113-119. | Dysphoric (n = 23) and nondysphoric (n = 24) individuals /  Emotional imagery task (presentation of emotion-involving images (pleasant, neutral, unpleasant) with instructions that participants imagine the scene as vividly as possible) | ANOVA results show no significant interaction between group and condition.  Groups differed across conditions with a **medium to large effect size (η^2^_p_ = .12).** |
| Lin, I. M., Tsai, Y. C., Peper, E., & Yen, C. F. (2013). Depressive mood and frontal alpha asymmetry during the luteal phase in premenstrual dysphoric disorder. Journal of Obstetrics and Gynaecology Research, 39(5), 998-1006. | Women with (n = 12) and without (n = 12) premenstrual dysphoric disorder (PMDD) /  Resting baseline, depressive induction & recall (thinking about an event that made them feel depressive), Recovery & Relaxation (neutral thoughts) | No calculation of interaction diagnosis*condition-  Significant differences between women with and without PMDD under both depressive induction and relaxation stages (Z = -2.31 and Z = -2,08 respectively, which at their sample size of n = 24 corresponds to **large effects (d = -1.06 and d = -0.93)** |
| Beeney, J. E., Levy, K. N., Gatzke-Kopp, L. M., & Hallquist, M. N. (2014). EEG asymmetry in borderline personality disorder and depression following rejection. Personality Disorders: Theory, Research, and Treatment, 5(2), 178. | Healthy controls (n = 21), participants with MD (n = 13) and participants with borderline PD /  Rejection task (Cyberball task, exclusion of participant in video game) | The interaction effect cannot be used for calculation, as it also encompassed the third BPD group.  The difference between healthy controls and MD participants specifically was significant after rejection. The study did not provide an effect size or means to calculate this, however when estimating means in both groups after rejection from the figure provided (MD: mean = -0.21, SD = 0.28; HC: mean = 0.05, SD = 0.27), a **large effect size can be estimated (d = 0.95)** |
| Messerotti Benvenuti, S., Buodo, G., Mennella, R., Dal Bò, E., & Palomba, D. (2019). Appetitive and aversive motivation in depression: The temporal dynamics of task-elicited asymmetries in alpha oscillations. Scientific Reports, 9(1), 17129. | Dysphoric (n = 23) and nondysphoric (n = 24) individuals /  Passive viewing of emotional pictures (pleasant, neutral, unpleasant) | Study did not analyze frontal alpha asymmetry, but rather event-related alpha power and is therefore not suitable for sample size calculation for our study. |
| Lopez‐Duran, N. L., Nusslock, R., George, C., & Kovacs, M. (2012). Frontal EEG asymmetry moderates the effects of stressful life events on internalizing symptoms in children at familial risk for depression. Psychophysiology, 49(4), 510-521. | Children 6-13 whose mothers had either a history of MD (n = 90) or no history of psychiatric conditions (n = 45) /  Watching of happy and sad film clips | No interaction was calculated.  High-risk participants displayed greater relative right lateral-frontal activation (F7/F8) compared to the low-risk children during the happy (**Cohen's d = -.47**) and sad (**d = -.50**) film clips in t-tests, showing **medium effect sizes**. |
| Stewart, J. L., Coan, J. A., Towers, D. N., & Allen, J. J. (2014). Resting and task‐elicited prefrontal EEG alpha asymmetry in depression: Support for the capability model. Psychophysiology, 51(5), 446-455. | Individuals with (n = 143) and without (n = 163) lifetime major depressive disorder (MDD) /  Participants made approach (angry / happy) and withdrawal (afraid / sad) facial expressions | There was no significant interaction between condition and group.  Groups **differed across conditions with a medium effect size (d = 0.62)** |

# B: Supplementary Tables

Supplementary Table 1: Demographics for current and remitted MD participants within the lifetime MD group

|  | Current MD (*n* = 9) | Remitted MD  (*n* = 25) |
| --- | --- | --- |
| Age in years (*M, SD*) | 21.67 (1.12) | 20.84 (1.72) |
| BDI-II score (*M, SD*) | 29.22 (7.69) | 8.80 (6.86) |
| ERQ reappraisal (*M, SD*) | 20.33 (8.35) | 24.36 (6.82) |
| Time since remission in weeks (*M, SD*) | - | 67.67 (83.06) |
| Current psychotherapy (n) | 4 | 6 |
| CBT (n) | 1 | 5 |
| Psychodynamic Psychotherapy (n) | 3 | 1 |
| Current medication | 4 | 6 |
| Laterality Index (FAA) Reappraise (*M, SD*) | -0.08 (0.23) | 0.04 (0.44) |
| Laterality Index (FAA) Attend (*M, SD*) | -0.04 (0.20) | 0.03 (0.43) |

Supplementary Table 2: Stimuli

| picture name | database | category |
| --- | --- | --- |
| 1603 | IAPS | positive |
| 1920 | IAPS | positive |
| 2040 | IAPS | positive |
| 2045 | IAPS | positive |
| 2070 | IAPS | positive |
| 2151 | IAPS | positive |
| 2156 | IAPS | positive |
| 2165 | IAPS | positive |
| 2205 | IAPS | negative |
| 2215 | IAPS | neutral |
| 2273 | IAPS | neutral |
| 2274 | IAPS | positive |
| 2311 | IAPS | positive |
| 2377 | IAPS | neutral |
| 2383 | IAPS | neutral |
| 2393 | IAPS | neutral |
| 2396 | IAPS | neutral |
| 2411 | IAPS | neutral |
| 2445 | IAPS | neutral |
| 2514 | IAPS | neutral |
| 2550 | IAPS | positive |
| 2595 | IAPS | neutral |
| 2635 | IAPS | neutral |
| 2688 | IAPS | negative |
| 2691 | IAPS | negative |
| 2710 | IAPS | negative |
| 3181 | IAPS | negative |
| 3185 | IAPS | negative |
| 3230 | IAPS | negative |
| 3530 | IAPS | negative |
| 5020 | IAPS | positive |
| 5199 | IAPS | positive |
| 5626 | IAPS | positive |
| 5830 | IAPS | positive |
| 5833 | IAPS | positive |
| 5910 | IAPS | positive |
| 6010 | IAPS | negative |
| 6150 | IAPS | neutral |
| 6211 | IAPS | negative |
| 6212 | IAPS | negative |
| 6312 | IAPS | negative |
| 6315 | IAPS | negative |
| 6540 | IAPS | negative |
| 6560 | IAPS | negative |
| 6563 | IAPS | negative |
| 6571 | IAPS | negative |
| 6825 | IAPS | negative |
| 7011 | IAPS | neutral |
| 7021 | IAPS | neutral |
| 7038 | IAPS | neutral |
| 7055 | IAPS | neutral |
| 7492 | IAPS | positive |
| 8420 | IAPS | positive |
| 8485 | IAPS | negative |
| 8540 | IAPS | positive |
| 9000 | IAPS | negative |
| 9145 | IAPS | negative |
| 9163 | IAPS | negative |
| 9185 | IAPS | negative |
| 9341 | IAPS | negative |
| 9403 | IAPS | negative |
| 9409 | IAPS | negative |
| 9415 | IAPS | negative |
| 9419 | IAPS | negative |
| 9423 | IAPS | negative |
| 9427 | IAPS | negative |
| 9435 | IAPS | negative |
| 9440 | IAPS | negative |
| 9490 | IAPS | negative |
| 9520 | IAPS | negative |
| 9560 | IAPS | negative |
| 9561 | IAPS | negative |
| 9599 | IAPS | negative |
| 9600 | IAPS | negative |
| 9610 | IAPS | negative |
| 9621 | IAPS | negative |
| 9810 | IAPS | negative |
| 9901 | IAPS | negative |
| 9902 | IAPS | negative |
| 9909 | IAPS | negative |
| 9920 | IAPS | negative |
| 9926 | IAPS | negative |
| 9930 | IAPS | negative |
| 2055.2 | IAPS | positive |
| 2345.1 | IAPS | negative |
| 2900.1 | IAPS | negative |
| D1 | BAPS | negative |
| D103 | BAPS | negative |
| D109 | BAPS | negative |
| D113 | BAPS | negative |
| D114 | BAPS | negative |
| D17 | BAPS | negative |
| D2 | BAPS | negative |
| D27 | BAPS | negative |
| D28 | BAPS | negative |
| D35 | BAPS | negative |
| D37 | BAPS | negative |
| D38 | BAPS | negative |
| D44 | BAPS | negative |
| D45 | BAPS | negative |
| D52 | BAPS | negative |
| D6 | BAPS | negative |
| D60 | BAPS | negative |
| D61 | BAPS | negative |
| D72 | BAPS | negative |
| D75 | BAPS | negative |
| D76 | BAPS | negative |
| D78 | BAPS | negative |
| D99 | BAPS | negative |
| J1 | BAPS | positive |
| J14 | BAPS | positive |
| J17 | BAPS | positive |
| J20 | BAPS | positive |
| J23 | BAPS | positive |
| J40 | BAPS | positive |
| J41 | BAPS | positive |
| J49 | BAPS | positive |
| J54 | BAPS | positive |
| J67 | BAPS | positive |
| J9 | BAPS | positive |
| N10 | BAPS | neutral |
| N12 | BAPS | neutral |
| N16 | BAPS | neutral |
| N19 | BAPS | neutral |
| N2 | BAPS | neutral |
| N20 | BAPS | neutral |
| N24 | BAPS | neutral |
| N28 | BAPS | neutral |
| N29 | BAPS | neutral |
| N30 | BAPS | neutral |
| N31 | BAPS | neutral |
| N33 | BAPS | neutral |
| N37 | BAPS | neutral |
| N39 | BAPS | neutral |
| N42 | BAPS | neutral |
| N45 | BAPS | neutral |
| N48 | BAPS | neutral |
| N57 | BAPS | neutral |
| N6 | BAPS | neutral |
| N63 | BAPS | neutral |
| R14 | BAPS | positive |
| R52 | BAPS | positive |
| R54 | BAPS | positive |
| R63 | BAPS | positive |

*Note.* IAPS = International Affective Picture System (Lang et al., 2008), BAPS = Besançon Attachment Picture System (Szymanska et al., 2019; Szymanska et al., 2015)

Supplementary Table 3: Number of included segments and percentage of EEG data removed due to artefacts in the main sample

|  | Lifetime MD | HC | Test | |
| --- | --- | --- | --- | --- |
|  | (*n* = 34) | (*n* = 25) | *t* | *p* |
| Negative-attend |  |  |  |  |
| Number of segments included (M, SD) | 176.76 (10.36) | 177.72 (8.00) | 0.384 | .702 |
| Data removed due to artefacts on average^1^ (in %) | 1.80% | 1.26% |  |  |
| Negative-reappraise |  |  |  |  |
| Number of segments included (M, SD) | 175.56 (12.50) | 175.96 (14.46) | 0.114 | .910 |
| Data removed due to artefacts on average^1^ (in %) | 2.46% | 2.24% |  |  |

^1^Average number of segments included / total number of segments

Supplementary Table 4: Number of included segments and percentage of EEG data removed due to artefacts in the validation sample

|  | Current MD | HC | Test | |
| --- | --- | --- | --- | --- |
|  | (*n* = 36) | (*n* = 38) | *t* | *p* |
| Negative-attend |  |  |  |  |
| Number of segments included (M, SD) | 161.82 (19.38) | 172.66 (10.69) | 2.952 | .005 |
| Data removed due to artefacts on average^1^ (in %) | 10.10% | 4.07% |  |  |
| Negative-reappraise |  |  |  |  |
| Number of segments included (M, SD) | 159.69 (23.06) | 172.03 (10.44) | 2.937 | .005 |
| Data removed due to artefacts on average^1^ (in %) | 11.28% | 4.42% |  |  |

^1^Average number of segments included / total number of segments

Supplementary Table 5: Means and standard errors of the laterality index (*M*, SE)

|  | Lifetime MD (*n* = 34) | HC (*n* = 25) |
| --- | --- | --- |
| Negative-attend | 0.014 (0.067) | 0.117 (0.092) |
| Negative-reappraise | 0.004 (0.067) | 0.103 (0.097) |

# C: Supplementary analyses

## Laterality index: All conditions

In the 2 (group) x 4 (condition) ANOVA examining the laterality index in all four conditions, there were no main effects of group (*F*(1, 57) = 0.849, *p* = .361, η^2^_p_ = 0.015) or condition (*F*(2.139, 121.920) = 0.387, *p* = 0.694, η^2^_p_ = 0.007) and no interaction between group*condition (*F*(2.139, 121.920) = 1.316, *p* = .272, η^2^_p_ = 0.023). Note that Greenhouse-Geisser correction was performed on the within-subject effect and within-between interaction, as sphericity was not met.

## Laterality index: Combined study samples

Regarding the explorative analysis with both the main sample of young adults with lifetime MD and the validation sample of adolescents with current MD, the 2 (MD vs. HC) x 2 (negative attend vs. negative reappraise) repeated measures ANOVA revealed no main effect of condition (*F*(1, 131) = 2.23, *p* = .138, η^2^_p_ = .017), group (*F*(1, 131) = 0.01, *p* = .904, η^2^_p_ < .001), and no interaction between group*condition (*F*(1, 131) = 1.38, *p* = .242, η^2^_p_ = .010).

## Behavioral results

The 2 (group) x 4 (condition) ANOVA on the SAM image ratings in all four conditions revealed a significant effect of condition (*F*(1, 54) = 279.751, *p* < .001, η^2^_p_ = 0.84). Following up the main effect of condition, post-hoc t-tests revealed that, across both groups, negative-reappraise images were rated more positively than negative-attend (*t*(55) = 13.951, *p* < .001), neutral images were rated more positively than negative-reappraise (*t*(55) = 4.424, *p* < .001), and positive images were rated more positively than neutral (*t*(55) = 13.852, *p* < .001). No other significant main effects or interactions emerged (all *ps* > .186).

# References

Belden, A. C., Pagliaccio, D., Murphy, E. R., Luby, J. L., & Barch, D. M. (2015). Neural activation during cognitive emotion regulation in previously depressed compared to healthy children: Evidence of specific alterations. *J. Am. Acad. Child Adolesc. Psychiatry*, *54*(9), 771-781. <https://doi.org/10.1016/j.jaac.2015.06.014>

Faul, F., Erdfelder, E., Buchner, A., & Lang, A.-G. (2009). Statistical power analyses using G*Power 3.1: Tests for correlation and regression analyses. *Behav. Res. Methods*, *41*(4), 1149-1160. <https://doi.org/10.3758/BRM.41.4.1149>

Faul, F., Erdfelder, E., Lang, A.-G., & Buchner, A. (2007). G*Power 3: A flexible statistical power analysis program for the social, behavioral, and biomedical sciences. *Behav. Res. Methods*, *39*(2), 175-191. <https://doi.org/10.3758/BF03193146>

Kudinova, A. Y., Owens, M., Burkhouse, K. L., Barretto, K. M., Bonanno, G. A., & Gibb, B. E. (2016). Differences in emotion modulation using cognitive reappraisal in individuals with and without suicidal ideation: An ERP study. *Cogn. Emot.*, *30*(5), 999-1007. <https://doi.org/10.1080/02699931.2015.1036841>

Lang, P. J., Bradley, M. M., & Cuthbert, B. N. (2008). *International affective picture system (IAPS): Affective ratings of pictures and instruction manual*. University of Florida.

Szymanska, M., Comte, A., Tio, G., Vidal, C., Monnin, J., Smith, C. C., Nezelof, S., & Vulliez-Coady, L. (2019). The Besançon affective picture set-adult (BAPS-Adult): Development and validation. *Psychiatry Res.*, *271*, 31-38. <https://doi.org/10.1016/j.psychres.2018.11.005>

Szymanska, M., Monnin, J., Noiret, N., Tio, G., Galdon, L., Laurent, E., Nezelof, S., & Vulliez-Coady, L. (2015). The Besançon Affective Picture Set-Adolescents (the BAPS-Ado): Development and validation. *Psychiatry Res.*, *228*(3), 576-584. <https://doi.org/10.1016/j.psychres.2015.04.055>

Yuan, J., Zhang, Y., Zhao, Y., Gao, K., Tan, S., & Zhang, D. (2023). The Emotion-Regulation Benefits of Implicit Reappraisal in Clinical Depression: Behavioral and Electrophysiological Evidence. *Neurosci. Bull.*, *39*(6), 973-983. <https://doi.org/10.1007/s12264-022-00973-z>
